# Fermented Cottonseed Meal as a Partial Replacement for Soybean Meal Could Improve the Growth Performance, Immunity and Antioxidant Properties, and Nutrient Digestibility by Altering the Gut Microbiota Profile of Weaned Piglets

**DOI:** 10.3389/fmicb.2021.734389

**Published:** 2021-09-01

**Authors:** Xueling Gu, Zhiqing Li, Jing Wang, Jiashun Chen, Qian Jiang, Nian Liu, Xiang Liu, Fan Zhang, Bie Tan, Hao Li, Xiaokang Ma

**Affiliations:** College of Animal Science and Technology, Hunan Agricultural University, Changsha, China

**Keywords:** fermented cottonseed meal, growth performance, immunity and antioxidant capacity, gut microbiota, weaned piglets

## Abstract

The study investigated the impact of fermented cottonseed meal (FCSM) on growth performance, immunity and antioxidant properties, nutrient digestibility, and gut microbiota of weaned piglets by replacing soybean meal with FCSM in the diet. The experimental piglets were fed with either the soybean meal diet (SBM group) or fermented cottonseed meal diet (FCSM group) for 14days after weaning. The digestibility of dry matter (DM), organic matter (OM), crude protein (CP), gross energy (GE), amino acids and nitrogen was higher in the FCSM diet than those in the SBM diet (*p*<0.05). The piglets in the FCSM group showed greater growth performance and lower diarrhea rate than those in the SBM group (*p*<0.05). The concentration of serum immunoglobulin G (IgG) and antioxidase, intestinal and hepatic antioxidase were increased and the concentration of malondialdehyde (MDA) in the serum was decreased in those piglets in the FCSM group compared to those piglets in the SBM group (*p*<0.05). The piglets in the FCSM group had a higher concentration of volatile fatty acids (VFAs) in their ileum and cecum and a higher Simpson index of ileum than piglets in the SBM group (*p*<0.05). The relative abundance of *Lactobacillus* and *[Ruminococcus]_torques_group* in ileum and *Intestinibacter*, *norank_f_Muribaculaceae*, *unclassified_o_Lactobacillales* and *[Eubacterium]_coprostanoligenes_group* in cecum were enhanced in piglets fed with the FCSM diet, whereas the relative abundance of *Sarcina* and *Terrisporobacter* were increased in piglets fed with the SBM diet. Overall, FCSM replacing SBM improved the growth performance, immunity and antioxidant properties, and nutrient digestibility; possibly *via* the alterant gut microbiota and its metabolism of weaned piglets.

**Graphical Abstract:**
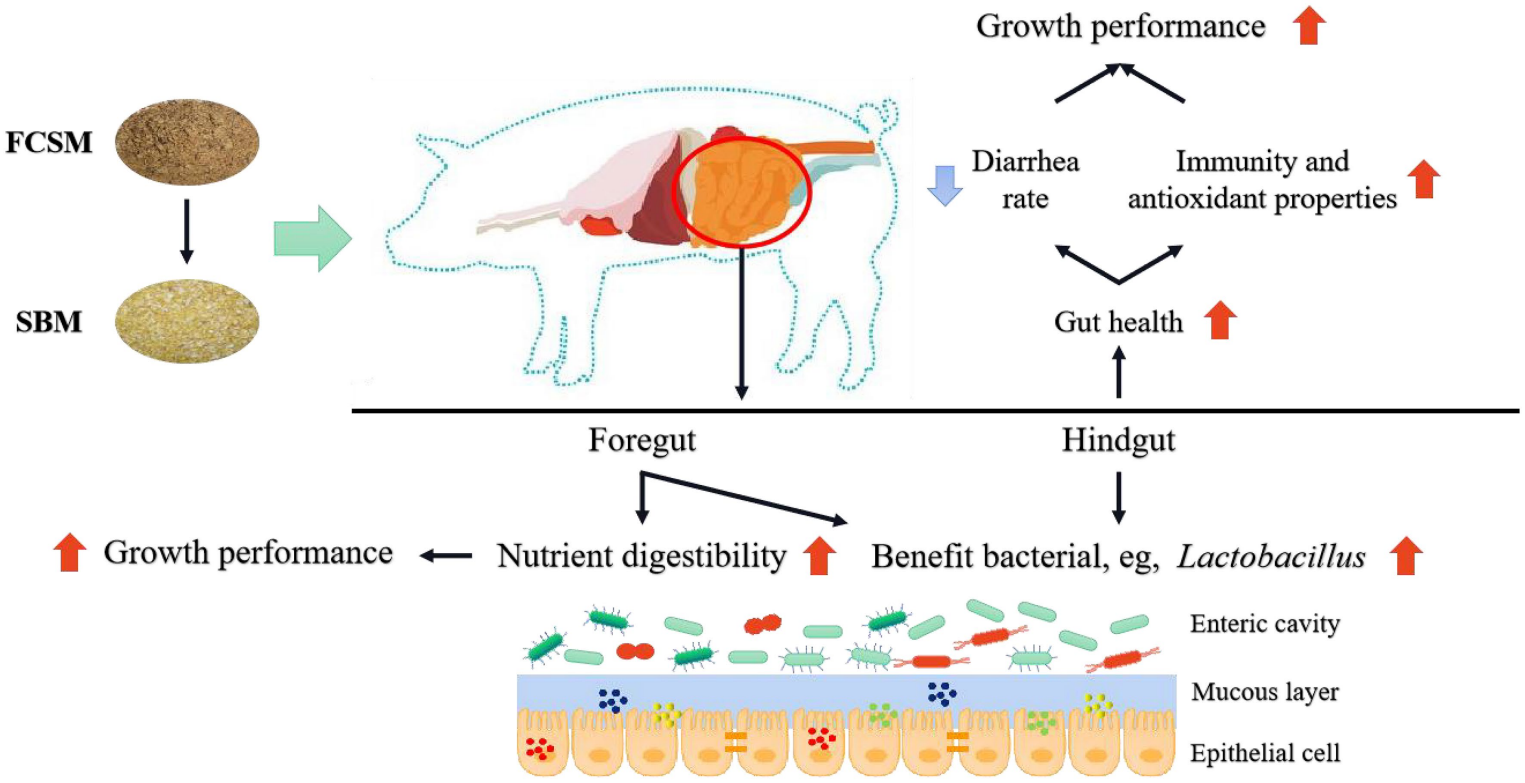
Fermented cottonseed meal as a partial replacement for soybean meal could improve the growth performance, immunity and antioxidant properties, and nutrient digestibility by altering the gut microbiota profile of weaned piglets. SBM, soybean meal; FCSM, fermented cottonseed meal.

Fermented cottonseed meal as a partial replacement for soybean meal could improve the growth performance, immunity and antioxidant properties, and nutrient digestibility by altering the gut microbiota profile of weaned piglets. SBM, soybean meal; FCSM, fermented cottonseed meal.

## Introduction

Soybean meal (SBM), an important protein source in the livestock industry, is highly recognized for its significant protein content and widespread availability (Azarm and Lee, 2014; Kim, 2014). However, the price of SBM has increased dramatically and the supply of high-quality protein feeding ingredients (such as SBM and fish meal) has been deficient in recent years. This leads to a higher livestock breeding expense and restricts the development of animal husbandry. Therefore, there is an urgent need for more alternative high-quality protein feeding ingredients to solve the protein source crisis in animal husbandry.

Cottonseed meal (CSM), a by-product obtained from the process of extracting the oil from cotton seed, is an attractive alternative protein source for livestock diets (Nagalakshmi et al., 2007). However, compared to SBM, the application of CSM as a feed ingredient in animal husbandry is limited due to the presence of anti-nutritional factors (Lordelo et al., 2007; Tang et al., 2012) such as free gossypol, cycloproponoic fatty acids, and crude fiber, which may cause negative effects on growth performance and organ functionality (Nie et al., 2015; Świątkiewicz, 2016). Fermented cottonseed meal (FCSM), a product produced by mixing solid CSM with liquid phases and then inoculating the mixture with beneficial microorganisms (Sun et al., 2014), can reduce free gossypol and improve the protein quality of CSM with solid state fermentation (Zhang et al., 2007). It has been suggested that the FCSM partial replacing SBM (about 6–8%) not only can improve the growth performance, immune and antioxidant capacity, and digestibility in broiler chickens (Sun et al., 2013; Nie et al., 2015; Wang et al., 2017; Niu et al., 2020), but also can reduce the F:G (the ratio of feed and gain weight) and diarrhea rate in nursery pigs, growing pigs and finishing pigs (Guan et al., 2017), which indicated that the FCSM has the potential to be a high-quality protein source.

Weaned piglet is a critical group in whole pig production, which consumes a large amount of high-quality protein ingredients. However, there is a lack of relevant research on the application of FCSM in weaned piglets. Therefore, the present study aimed to compare the effects of dietary SBM and FCSM on growth performance, immunity and antioxidant properties, nutrient digestibility, and gut microbiota of weaned piglets.

## Materials and Methods

The animal handling and all procedures of this study received approval from the Animal Care and Use Ethics Committee of the Hunan Agricultural University (Changsha). The FCSM used in this experiment was provided by the Tycoon Group (Xinjiang, China). The SBM used in this experiment was provided by Hunan Lifeng Biological Technology Co., Ltd. (Hunan, China). Table 1 shows the nutrient composition of SBM and FCSM.

**Table 1:** Analyzed composition of the fermented cottonseed meal and soybean meal (%, as-fed basis).

Item	SBM	FCSM
Dry matter	89.95	93.06
Crude protein	45.11	50.20
Ether extract	1.92	1.36
Neutral detergent fiber	13.86	19.45
Acid detergent fiber	9.75	11.70
Ash	6.21	6.65
Calcium	0.35	0.37
Phosphorus	0.69	0.98
GE, MJ/kg	17.55	17.81
*Indispensable AA*		
Arginine	3.36	6.09
Histidine	1.28	1.59
Isoleucine	1.89	1.52
Leucine	3.29	2.84
Lysine	2.86	2.36
Methionine	0.63	0.67
Phenylalanine	2.20	2.81
Threonine	1.72	1.78
Tryptophan	0.45	0.63
Valine	2.06	2.14
*Dispensable amino acids*		
Alanine	2.16	1.97
Aspartate	5.21	4.88
Cystine	0.61	0.79
Glutamine	7.34	10.05
Glycine	1.89	1.92
Proline	2.06	2.46
Serine	2.31	2.25
Tyrosine	1.68	1.49

Analysis conducted in duplicates. SBM, soybean meal; FCSM, fermented cottonseed meal.

### Animal Treatment and Experimental Design

A total of 32 Duorc×(Landrace×Yorkshire) growing barrows, with an average initial body weight (BW) of 7.85±0.49kg, were allotted to two dietary treatments in a completely randomized design with 16 repetitions per treatment according to their body weight. The dietary treatments include SBM diet and FCSM diet: the FCSM diet was formulated by adding 6% FCSM to replace the SBM compared with the SBM diet, and corn and soy oil was changed to balance the energy and protein levels (Table 2). The experimental diets and vitamin-mineral premix were configured to meet the nutritional needs of nursery piglets as recommended by the NRC (2012). The experimental period lasted for 14days. The house, feed trough and drinker were thoroughly cleaned and disinfected before starting the experiment. The temperature of the pig house was kept at 24–28°C, and the relative humidity was controlled at 60–70%. All the pigs were provided *ad libitum* access to water and feed. The daily feed intake and BW of each pig were recorded on day 0 and day 14 to calculate the average daily gain (ADG), average daily feed intake (ADFI) and feed conversion rate (ADG:ADFI, G:F). A scoring system was applied to indicate the presence and severity of diarrhea as following: 1=hard feces; 2=slightly soft feces; 3=soft, partially formed feces; 4=loose, semiliquid feces; and 5=watery, mucous-like feces.

**Table 2:** Ingredients composition and nutrient levels of the experimental diets (%, as-fed basis).

Ingredients	SBM	FCSM
Corn	42.02	41.55
Soybean meal	20.00	14.00
Extruded full-fat soybean	12.00	12.00
Soy protein concentrate	4.00	4.00
Fermented cottonseed meal	0.00	6.00
Whey powder	8.00	8.00
Soy oil	4.61	5.02
Sucrose	5.38	5.38
Dicalcium phosphate	1.26	1.29
Limestone	0.99	0.98
Salt	0.30	0.30
Lysine	0.41	0.45
Methionine	0.13	0.13
Threonine	0.13	0.13
Tryptophan	0.02	0.02
Chromic oxide	0.25	0.25
Vitamin-mineral premix^1^, no antibiotic	0.50	0.50
*Calculated nutrient levels*		
Metabolized energy, kcal/kg	3,400	3,400
Crude protein	20.04	20.32
Standardized ileal digestible lysine	1.35	1.35
Standardized ileal digestible methionine	0.39	0.39
Standardized ileal digestible threonine	0.79	0.79
Standardized ileal digestible tryptophan	0.22	0.22

1 The components and contents of the premix providing nutrients for per kg feed are as follows: Vitamin A, 12,000IU; Vitamin D3, 2,500IU; Vitamin E, 30IU; Vitamin K3, 30mg; Vitamin B12, 12μg; Riboflavin, 4mg; Pantothenic acid, 15mg; Niacin, 40mg; Choline chloride, 400mg; Folic acid, 0.7mg; Vitamin B1, 1.5mg; Vitamin B6, 3mg; Biotin, 0.1mg; Manganese, 40mg; Iron, 90mg; Zinc, 100mg; Copper, 8.8mg; Iodine, 0.35mg; Selenium, 0.3mg.

SBM, soybean meal; FCSM, fermented cottonseed meal.

### Sample Collection

By the end of the experiment, six samples of blood (10ml) were collected from the precaval veins of each group of piglets after fasting for 12h. After standing the blood samples for 1h at 4°C, they were centrifuged at 3,000×*g* for 15min at 4°C, whereupon the serum samples obtained were immediately stored at −80°C for immunoglobulin and antioxidant indices analysis including immunoglobulin A (IgA), immunoglobulin G (IgG), immunoglobulin M (IgM), superoxide dismutase (SOD), glutathione peroxidase (GSH-Px), total antioxidant capacity (T-AOC), and malondialdehyde (MDA). After the blood collection, six piglets closest to the average BW from each group were slaughtered, and then the jejunum, ileum and liver, were sampled through a sterile laparotomy, which was collected in centrifuge tubes and immediately placed in liquid nitrogen and then stored at the temperature of −80°C for analysis of antioxidant indices levels of SOD, GSH-Px, catalase (CAT), T-AOC and MDA in jejunal, ileal and liver tissue. Besides, the ileum and cecum segments were isolated to collect the digesta samples using centrifuge tubes and a part of them immediately placed in liquid nitrogen and then stored at the temperature of −80°C for analysis of microbiome and metabolite. The rest of samples were stored at −20°C for subsequent chemical composition analysis to calculate nutrient digestibility.

### Determination of the Digestibility of Nutrients and Amino Acids

The feed samples and ileal digesta after freeze-drying were weighed in parallel samples for analysis and determination. Dry matter (DM), organic matter (OM), crude protein (CP), and gross energy (GE) contents were determined following the AOAC (2006) procedures. The amino acid (AA) profiles were detected by High Performance Liquid Chromatography (HPLC; Agilent 1200, Agilent Technologies, United States). Lysine and threonine were detected after hydrolyzing with 6mol/L HCl at 105°C for 24h. Methionine was analyzed as methionine sulfone after cold performic acid oxidation overnight before hydrolysis. Tryptophan was determined after hydrolyzing with 4mol/L LiOH at 110°C for 20h. The contents of acid insoluble ash (AIA) were determined in according to the method of Keulen and Young (1977) and the apparent ileal digestibility (AID) of amino acids and nitrogen was calculated as described by Stein et al. (2007).

### Immunoglobulin and Antioxidant Indices Analysis

Frozen jejunal, ileal and liver tissue (2mg) in 2ml of phosphate-buffered saline was homogenized on ice with an Ultra-Turrax homogenizer (Bioblock Scientific, Illkirch, France) for 10s at 6,800rpm. The homogenate was centrifuged at 950×*g* for 10min at 4°C, and the supernatant was stored in a 2-ml centrifuge tube at −80°C until analysis. The GSH-Px and CAT activities, T-AOC, and MDA concentrations in the jejunal, ileal and liver tissue and serum were assayed using a UV/visible spectrophotometer (UV-2450; Shimadzu, Kyoto, Japan). The assays were conducted using assay kits purchased from Nanjing Jiancheng Institute of Bioengineering (Nanjing, Jiangsu, China) and conducted according to the manufacturer’s instructions. All samples were measured in triplicate, at appropriate dilutions, and the activities of the enzymes were estimated from the linear range of standard curves constructed with the pure enzymes. The protein concentration of the supernatants was determined using Coomassie Brilliant Blue G250 (BlueGene, Shanghai, China).

### Analysis for VFAs Using a Gas Chromatographic Method

The concentration of volatile fatty acids (VFAs) including short chain fatty acids (SCFAs) and branched chain fatty acids (BCFAs) in digesta were analyzed using a gas chromatographic method. Briefly, approximately 1.0g of digesta samples were first homogenized in the 1.5ml deionized water. After being centrifuged at 15,000×*g* at 4°C for 10min, supernatants (1ml of each) were acidified with 25% metaphosphoric acid at a 1:5 ratio (1 volume of acid for 5 volumes of sample) for 30min while on ice. The sample was injected into a GC 2010 series gas chromatograph (Shimadzu, Japan) equipped with a CP-Wax 52 CB column 30.0m×0.53mm i.d (Chrompack, Netherlands). The injector and detector temperatures were 75 and 280°C, respectively. All procedures were performed in triplicate and total VFAs were determined as the sum of analyzed SCFAs (acetate, propionate, butyrate, and valerate) and BCFAs (isobutyrate and isovalerate).

### Analysis for Bacterial Microbiota by 16S RNA

Total genomic DNA of 12 digesta samples were extracted using a Stool DNA Isolation Kit (Tiangen Biotech Co., Ltd., Beijing, China) following the manufacturer’s instructions. The quantity and quality of extracted DNAs were measured using a NanoDrop ND-1000 spectrophotometer (Thermo Fisher Scientific, United States) and agarose gel electrophoresis, respectively. The genes of bacteria 16S ribosomal RNA in the region of V4–V5 were amplified by using polymerase chain reaction (PCR) with primers (515F 5'-barcode- GTGCCAGCMGCCGCGG)-3' and (907R 5'-120CCGTCAATTCMTTTRAGTTT-3'). Electrophoresis was applied to analyze the integrity of PCR amplicons by using a Tapestation Instruction (Agilent technologies, United States). AxyPrep DNA Gel 122 Extraction Kit was chosen to extract and purify PCR amplicons using 2% agarose gels (Axygen 123Biosciences, Union City, CA, United States) and then the production was quantified using QuantiFluor™ -ST and sequenced on an Illumina MiSeq system. QIIME software was used to demultiplex and quality-filtered raw Illumina fastq files. Operational taxonomic units (OTUs) were defined as a similarity threshold of 0.97 using UPARSE. Then UCHIME was applied to identify and delete the abnormal gene sequences. RDP database^1^ was also referenced to take the taxonomy-based analysis for OUTs using RDP classifier at a 90% confidence level. The *α*-diversity indices including Simpson and Chao1 were analyzed by Mothur v.1.30.2. Principal co-ordinates analysis (PCoA) tools in R language were used for PCoA. The histogram of linear discriminant analysis (LDA) distribution was implemented using LDA effect size analysis (LEfSe) software.

### Statistical Analysis

All data were analyzed by the GLM procedure of SPSS 21.0 (SPSS Inc., Chicago, IL, United States), and each piglet was regarded as a statistical unit. Data are showed as Mean values with standard error of the total mean (SEM). For all tests, *p*<0.05 was considered as significant difference, while 0.05<*p*<0.10 as a tendency.

## Results

### Growth Performance

Over the experimental period, it has been noticed that piglets in the FCSM treatment had higher the final BW, ADG, and G:F (*p*<0.05) and lower diarrhea incidence compared to those in the SBM treatment (*p*<0.05; Table 3). No difference was observed in ADFI between the two dietary treatments (Table 3).

**Table 3:** Effects of two protein source on performance of weaned piglets.

Items	SBM	FCSM	SEM	*p*
Initial BW, kg	7.79	7.78	0.04	0.89
Final BW, kg	11.68	12.04	0.08	0.03
ADG, g	277.86	304.29	7.12	0.04
ADFI, g	441.05	454.16	9.17	0.46
G:F	0.63	0.67	0.01	0.03
Diarrhea incidence, %	4.78	2.42	0.47	0.02

Data were shown as the mean with the SEM (*n*=16). BW, body weight; ADG, average daily gain; ADFI, average daily feed intake; G:F, ADG:ADFI; SBM, soybean meal; FCSM, fermented cottonseed meal.

### Nutrient Digestibility

As shown in Table 4, the FCSM diet had higher apparent total tract digestibility and ileal digestibility of nutrients in terms of DM, OM, CP, and GE than the SBM diet (*p*<0.05). Moreover, higher AID of essential AA including histidine, isoleucine, leucine, phenylalanine, valine, and nonessential AA including asparagine were discovered in the FCSM group compared to that in the SBM group (*p*<0.05, Table 5). Furthermore, the AID of total nitrogen was enhanced in the FCSM group in comparison with the SBM group (*p*<0.05).

**Table 4:** Effects of two protein source on apparent total tract digestibility and ileal digestibility of nutrients of weaned piglets (%).

Item	SBM	FCSM	SEM	*p*
*Apparent total tract digestibility of nutrients*				
DM	84.24	85.55	0.22	0.01
OM	86.08	87.53	0.23	0.01
CP	77.81	80.08	0.23	0.01
GE	84.01	85.35	0.22	0.01
*Apparent ileal digestibility of nutrients*				
DM	86.24	87.85	0.22	0.01
OM	87.88	89.83	0.23	0.01
CP	79.41	82.18	0.23	0.01
GE	85.40	87.55	0.22	0.01

Data were shown as the mean with the SEM (*n*=6). DM, dry matter; OM, organic matter; CP, crude protein; GE, gross energy; SBM, soybean meal; FCSM, fermented cottonseed meal.

**Table 5:** Effects of two protein source on apparent ileal digestibility of amino acids and nitrogen of weaned piglets (%).

Items	SBM	FCSM	SEM	*p*
*Essential AA (%)*				
Arginine	84.39	89.10	2.64	0.25
Histidine	93.01	96.48	0.77	0.02
Isoleucine	75.80	82.45	1.69	0.03
Leucine	75.45	81.22	1.43	0.02
Lysine	90.50	93.21	1.21	0.16
Methionine	90.84	93.12	1.16	0.21
Phenylalanine	94.06	96.26	0.62	0.04
Threonine	77.21	78.65	0.51	0.09
Tryptophan	78.38	79.06	1.03	0.65
Valine	80.60	86.79	1.34	0.01
*Nonessential AA (%)*				
Alanine	71.55	75.48	1.50	0.11
Asparagine	66.16	72.31	1.51	0.02
Cystine	61.97	63.49	2.74	0.71
Glutamine	86.67	89.86	1.57	0.20
Glycine	63.56	64.73	1.17	0.50
Proline	85.28	85.28	1.79	0.99
Serine	80.98	82.85	1.42	0.39
Tyrosine	75.73	81.27	1.85	0.07
Total nitrogen (%)	79.41	82.18	0.22	< 0.01

Data were shown as the mean with the SEM (*n*=6). AA, amino acid; SBM, soybean meal; FCSM, fermented cottonseed meal.

### Immunity and Antioxidant Properties

The results have shown that piglets in the FCSM group had higher serum IgG (*p*<0.05) than those in the SBM group (Table 6). However, no significant difference was observed in terms of serum IgA and IgM (*p*>0.05) of weaned piglets between the two groups. The effects of FCSM and SBM on serum, intestine and liver antioxidant enzyme activity and oxidant products on weaned piglets has been shown in Table 7. Compared with the SBM group, the SOD and GSH-Px in serum, jejunum, and ileum were improved on the piglets in the FCSM group (*p*<0.05). Moreover, the jejunal T-AOC and the liver GSH-Px were enhanced on the piglets in the FCSM group (*p*<0.05). Furthermore, the serum MDA was reduced on the piglets in the FCSM group (*p*<0.05).

**Table 6:** Effects of two protein source on serum immune of weaned piglets (%).

Items	SBM	FCSM	SEM	*p*
IgG (g/L)	7.82	9.27	0.38	0.03
IgA (g/L)	1.07	1.06	0.04	0.86
IgM (g/L)	0.84	0.91	0.06	0.59

Data were shown as the mean with the SEM (*n*=6). IgG, immunoglobulin G; IgA, immunoglobulin A; IgM, immunoglobulin M; SBM, soybean meal; FCSM, fermented cottonseed meal.

**Table 7:** Effects of two protein source on serumal, intestinal and hepatic antioxidant enzyme activity and oxidant products of weaned piglets.

Items	SBM	FCSM	SEM	*p*
*Serum*				
SOD (U/ml)	110.38	142.56	4.25	0.03
GSH-Px (U/ml)	724.89	796.57	18.26	0.02
T-AOC (U/ml)	7.38	8.44	0.54	0.21
MDA (nmol/ml)	4.92	2.87	0.32	0.02
*Jejunum*				
SOD (U/mg prot)	129.51	206.25	10.30	0.01
GSH-Px (U/mg prot)	248.40	361.90	15.75	0.01
CAT (U/mg prot)	45.43	44.33	2.08	0.72
T-AOC (U/mg prot)	54.05	81.28	4.12	0.01
MDA (nmol/mg prot)	3.92	3.17	0.66	0.45
*Ileum*				
SOD (U/mg prot)	144.49	184.88	6.06	0.01
GSH-Px (U/mg prot)	275.33	394.29	28.60	0.02
CAT (U/mg prot)	47.23	39.27	3.86	0.19
T-AOC (U/mg prot)	45.06	60.60	6.03	0.11
MDA (nmol/mg prot)	5.31	4.60	0.43	0.29
*Liver*				
SOD (U/mg prot)	27.65	34.88	3.47	0.18
GSH-Px (U/mg prot)	96.34	124.23	6.93	0.02
CAT (U/mg prot)	9.13	11.81	1.53	0.26
T-AOC (U/mg prot)	26.09	31.18	3.42	0.33
MDA (nmol/mg prot)	4.38	3.21	0.46	0.11

Data were shown as the mean with the SEM (*n*=6). SOD, superoxide dismutase; GSH-Px, glutathione peroxidase; CAT, catalase; T-AOC, total antioxidant capacity; MDA, malondialdehyde; SBM, soybean meal; FCSM, fermented cottonseed meal.

### VFAs Composition

The VFAs composition in ileal and cecal digesta of weaned piglets were obtained in Table 8. Compared with the SBM group, the concentration of acetate, propionate, butyrate, isobutyrate, isovalerate, and total VFAs in ileal digesta of weaned piglets were increased in the FCSM group (*p*<0.05). Similarly, the concentration of acetate, propionate, valerate, isobutyrate, isovalerate, and total VFAs in cecal digesta of weaned piglets were higher (*p*<0.05) and the concentration of butyrate had a tendency to increase in the FCSM group (0.05<*p*<0.1).

**Table 8:** Effects of protein source on volatile fatty acids composition in ileal and cecal digesta of weaned pigs (mg/g digesta).

Items	SBM	FCSM	SEM	*p*
*Ileum*				
Acetate	0.70	0.83	0.02	0.01
Propionate	0.35	0.42	0.01	0.01
Butyrate	0.31	0.40	0.01	<0.01
Valerate	0.26	0.26	0.01	0.47
Isobutyrate	0.09	0.05	0.01	<0.01
Isovalerate	0.22	0.17	0.01	<0.01
Total VFAs^1^	1.93	2.13	0.01	<0.01
*Cecum*				
Acetate	3.79	4.43	0.07	<0.01
Propionate	2.72	3.36	0.18	0.04
Butyrate	1.77	2.03	0.09	0.07
Valerate	0.75	0.99	0.05	0.02
Isobutyrate	0.41	0.31	0.02	0.01
Isovalerate	0.61	0.47	0.02	0.01
Total VFAs^1^	10.06	11.60	0.27	0.01

1 Total VFAs=Acetate+Propionate+Butyrate+Valerate+Isobutyrate+Isovalerate.

Data were shown as the mean with the SEM (*n*=6). VFAs, volatile fatty acids; SBM, soybean meal; FCSM, fermented cottonseed meal.

### Gut Microbiota Diversity

**Figure 1:**
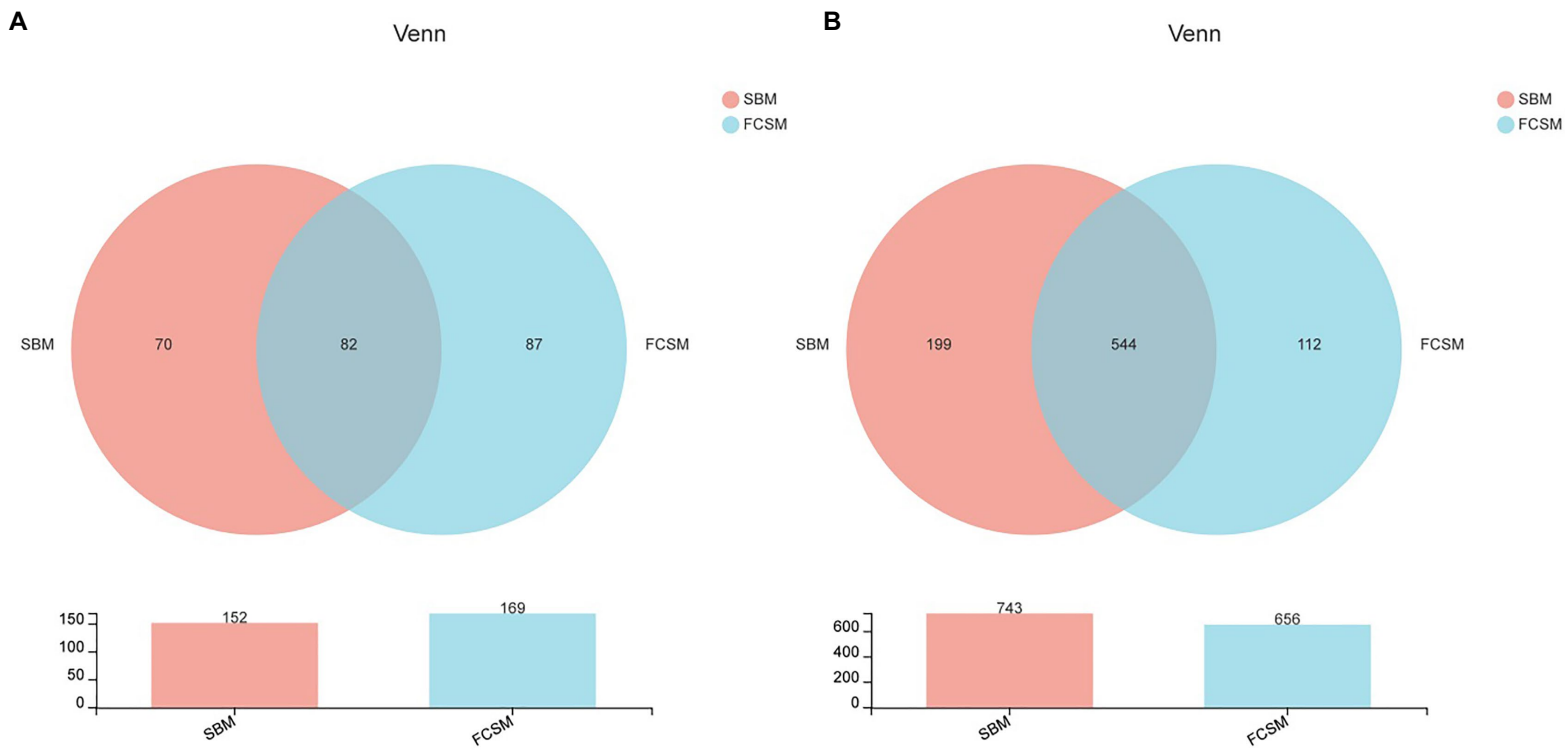
Effects of two protein sources on the ileac **(A)** and cecal **(B)** microbes at the operational taxonomic unit (OTU) in piglets. The individual minipig was regarded as the experimental unit (*n*=6). SBM, soybean meal; FCSM, fermented cottonseed meal.

**Figure 2:**
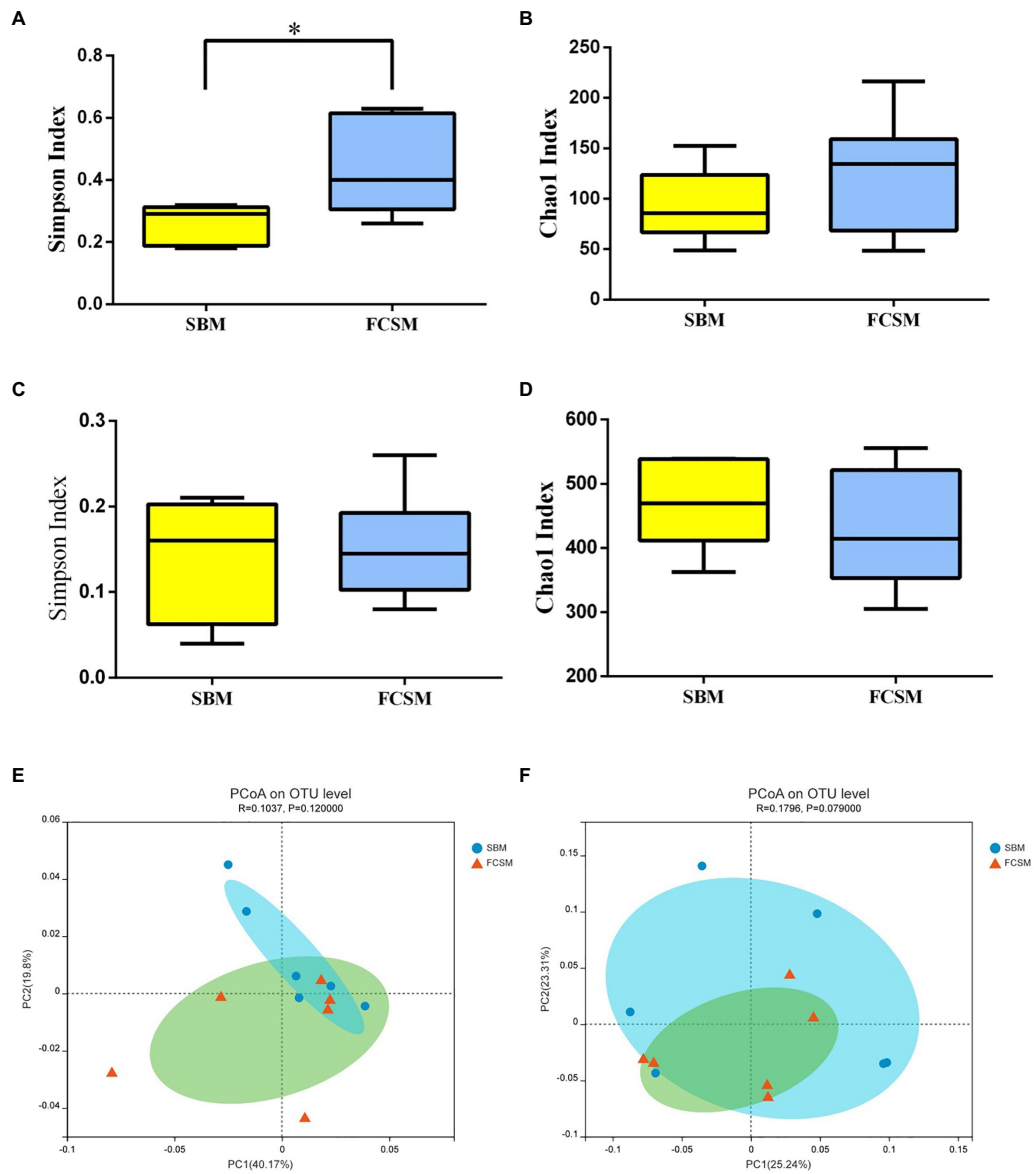
Effects of two protein sources on the *α*-diversity indexes including Simpson index of ileac **(A)** and cecal **(C)** microbes, Chao1 index of the ileac **(B)** and cecal **(D)** microbes, *β*-diversity of the ileac **(E)** and cecal **(F)** microbes in piglets. The individual minipig was regarded as the experimental unit (*n*=6). SBM, soybean meal; FCSM, fermented cottonseed meal. ^*^p < 0.05.

The OTUs of the ileal digesta from SBM and FCSM groups were 152 and 169, respectively, among which 82 common OTUs have been identified (Figure 1A). On the other hand, the OTUs of the cecal digesta from SBM and FCSM groups were 743 and 656, respectively, wherein 544 common OTUs have been identified (Figure 1B). The *α*-diversity of ileal and cecal microbiota including Simpson index and Chao1 index were presented in Figures 2A–D. The FCSM diet induced higher Simpson index of ileum than the SBM (*p*<0.05), whereas no difference was shown in the other index between the two dietary treatments neither in the ileum nor cecum (*p*>0.05). The *β*-diversity of bacterial community between SBM and FCSM was presented with PCoA (Figures 2E,F), showing a tendency of different clustering of microbial communities in cecum (0.05<*p*<0.1, Figure 2F).

**Figure 3:**
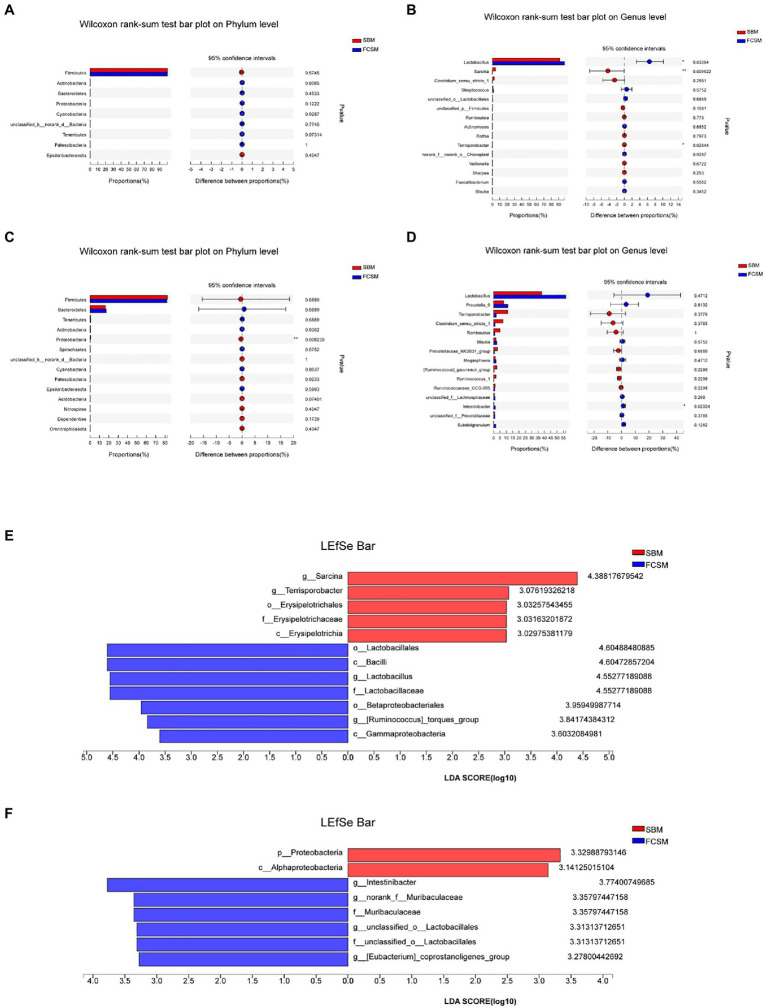
Different microbiota comparison by the student *t*-test on Phylum and Genus of ileum **(A,B)** and cecum **(C,D)** was shown in **(A–D)**. LDA effect size analysis (LEfSe) analysis showed significantly changed bacteria between SBM and FCSM group in ileum **(E)** and cecum **(F)**. The individual minipig was regarded as the experimental unit (*n*=6). SBM, soybean meal; FCSM, fermented cottonseed meal.

At the phylum level, Firmicutes and Bacteroidetes were the dominant bacteria in both ileum and cecum. There was no difference found by the students’ tests in microbiota at the phylum level between the two treatment groups in ileum (*p*>0.05; Figure 3A). However, the proportion of cecal Proteobacteria was decreased (*p*<0.05) in the FCSM group compared with the SBM group (Figure 3C). Besides, at the genus level (Figures 3B,D), the proportion of ileal *Lactobacillus* was enhanced but the ileal *unclassified_p_Firmicutes* and cecal *Ruminococcus_1* was decreased in the piglets in the FCSM group rather than that in the SBM group (*p*<0.05).

The LEfSe analysis was used to identify the significantly different bacteria in the ileum and cecum between the two treatment groups from the phylum to genus level (Figures 3E,F). The relative abundance of *Lactobacillus* and *[Ruminococcus]_torques_group* in ileum and *Intestinibacter*, *norank_f_Muribaculaceae*, *unclassified_o_Lactobacillales* and *[Eubacterium]_coprostanoligenes_group* in cecum were enhanced in piglets fed with the FCSM diet than those fed with SBM diet, whereas the relative abundance of *Sarcina* and *Terrisporobacter* in ileum were decreased in piglets fed with the FCSM diet.

## Discussion

Cottonseed meal has not been widely used because it contains a large number of anti-nutritional factors, which result negative effects on growth performance, immune and antioxidant capacity and nutrient digestibility in animals (Nagalakshmi et al., 2007; Nie et al., 2015; Świątkiewicz, 2016). After going through solid state fermentation, FCSM has much less free gossypol and other anti-nutritional factors, and definitively improved protein quality (Zhang et al., 2006, 2007; Sun et al., 2014; Wang et al., 2017). Sun et al. (2013) reported that the appropriate inclusion of FCSM replacing SBM improved the growth of yellow-feathered broiler chickens. Similarly, FCSM supplementation improved the ADG and G:F ratio of yellow-feathered broilers from the 43rd to 64th and the 21st to 64th day, respectively (Nie et al., 2015), which indicated that FCSM is beneficial for broilers as it enhanced their growth performance and digestion. Consistently, the current study showed that the piglets in the FCSM group presented greater growth performance and a lower diarrhea rate than those in the SBM group. The fermentation process of CSM might be one of the reasons, which effectively decreases free gossypol level, and increases acid-soluble protein level in CSM, and therefore further improves the digestive enzyme activity and nutrient digestibility in weaned piglets (Sun et al., 2014; Wang et al., 2017). This explanation has been confirmed by the nutrient digestibility of weaned piglets in FCSM and SBM treatments. The present results demonstrated that the apparent total tract digestibility and ileal digestibility of nutrients, essential and nonessential AA have been enhanced in piglets within the FCSM group.

Moreover, the microbial fermentation process can produce many beneficial substances, such as small-size peptides, exoenzymes, vitamins, organic acids, which can promote the immunity of animals (Johnson, 2013; Zhao et al., 2016). A study have reported that fermentation with *Bacillus subtilis BJ-1* can reduce free gossypol level in CSM and dietary inclusion of 12% FCSM can improve immunity (Tang et al., 2012). Wang et al. (2017) reported that the dietary supplementation of FCSM increased serum IgM and IgG levels compared with SBM groups in broilers. In the present study, the concentration of serumal IgG was increased in piglets in the FCSM group in comparison with those in the SBM group, which might be due to the live microbes in FCSM acting as probiotics to enhance the humoral immune response of the animals (Latesh et al., 2013; Stašová et al., 2015).

The immunity of piglets is highly related to antioxidant capacity (Liu et al., 2021). Besides, antioxidase can improve the immunity by promoting the bacterial clearance and regulates phagocyte numbers (Peterman et al., 2015). In addition, a microbial FCSM increased the antioxidant activity in diets for Nile tilapia (Lim and Lee, 2011). Similarly, the present study showed that the antioxidation-related enzymes in serum, jejunum, ileum and liver were improved in piglets in the FCSM group compared with that in the SBM group. Additionally, the serum MDA was reduced in piglets in the FCSM group, which indicated that FCSM improved the antioxidative abilities compared with SBM (Wang et al., 2017). This may be one of the reasons that the improvement of growth performance in piglets has found in the FCSM group (Ma, X. et al., 2019; Ma, X. K. et al., 2019).

Many studies suggest that food or feeds fermented by probiotics may be potentially an effective strategy to regulate the gut microbiota and its metabolites (Azad et al., 2020; Niu et al., 2020; Gu et al., 2021; Li et al., 2021). The VFAs, especially butyric acid, as a microbial metabolite, can not only improve the growth performance, but also boost the immunity of piglets (Fang et al., 2014; Zhao et al., 2018). Zhao et al. (2018) reported that dietary fiber increases the butyrate-producing bacteria and improves growth performance of weaned piglets. Tsai et al. (2019) discovered that feeding sodium butyrate during the nursery phase tended to alter blood cell count and improve growth performance of weaned pigs. In the present study, the VFAs of ileum and cecum were increased in the FCSM group than that in the SBM group. This may explain the improved growth performance of piglets. Moreover, VFAs, as important intermediate products during anaerobic digestion, may influence the fermentation characteristics of hindgut (Jha and Berrocoso, 2016; Seradj et al., 2018). The protein fermentation metabolites in the hindgut are amines, SCFAs and BCFAs, among which amines must be converted from nitrogen-containing groups, while BCFAs are only produced from the fermentation of three branched chain amino acids, leucine, isoleucine and valine (Jha and Berrocoso, 2016; Seradj et al., 2018). Over-fermentation of protein in the hindgut is an important cause of diarrhea in piglets. The current study found that FCSM replacing SBM decreased the isobutyrate and isovalerate of the cecum, suggesting that FCSM reduced hindgut fermentation, which may be responsible for the reduced diarrhea rate in piglets.

In recent years, the interaction and connections between dietary protein, gut microbe and host has received increasing attention. Segmented exogenous microbiota transplantation proved the spatial heterogeneity of bacterial colonization along the gastrointestinal tract, i.e., the microbiota from one specific location selectively colonizes its homologous gut region (Li et al., 2020). The number of microorganisms in the hindgut was higher than that in the foregut (Jamet et al., 2011), which is consistent with the result in the present study. It has been previously shown that FCSM enhanced the Simpson index of ileum in piglets, which has a great contribution towards the improvement of intestinal health and maturation in piglets (Wang, X. et al., 2019). Lower protein concentration or better protein sources in the diets can improve hindgut health by preventing the proliferation of pathogenic bacteria and reduced the risk of colitis (Vidal-Lletjós et al., 2017; Najafabadi et al., 2019). Studies have shown that protein fermentation can change the composition and function of intestinal flora (Lu et al., 2019; Wang, H. et al., 2019). Consistently, in the present study, the analysis of the PCoA has shown that the microbial composition between the SBM and FCSM groups are slightly different.

Firmicutes and Bacteroidetes were the most dominant phyla in the pig (Chen et al., 2017). In our study, Firmicutes is the most dominant phyla in ileum and Firmicutes and Bacteroidetes in cecum. *Lactobacillus*, as a potential probiotic, possesses the resistance to pathogen, anti-inflammatory and antioxidant capacity, and ability to improve of gut microbiota profile (He et al., 2019; Yang et al., 2020). Wang et al. (2017) has discovered that *Lactobacilli* and total anaerobic bacteria counts in ceca digesta of birds fed FCSM were improved compared with birds fed CSM on days 21 and 42. Likewise, the FCSM replacing the SBM has also enhanced the relative abundance of *Lactobacillus* of ileum and cecum in the present study. He et al. (2019) has found that *Lactobacillus johnsonii L531* reduced pathogen load and helped maintain SCFA levels in the intestine of pigs challenged with *Salmonella enterica Infantis*. Therefore, this might be one of the reasons that piglets in the FCSM group had higher level of VFAs than that in the SBM group. On the other hand, proteobacteria is the largest phylum of bacteria, including many pathogenic bacteria, such as *Escherichia coli*, *Salmonella*, *Vibrio cholerae*, *Helicobacter pylori* and other well-known species. In the current study, the relative abundance of Proteobacteria was decreased in piglets in the FCSM group, suggesting that the FCSM has the potential function to inhibit harmful bacteria, and improves the gut microbiota profile than SBM (Wang et al., 2017). In conclusion, FCSM replacing SBM improved the growth performance, immunity and antioxidant properties, nutrients digestibility possibly *via* the altering gut microbiota profile and its metabolites in weaned piglets.

## Data Availability Statement

The data presented in the study are deposited in the (NCBI SRA) repository, accession number (PRJNA743130).

## Ethics Statement

The animal study was reviewed and approved by Animal Care and Use Ethics Committee of the Hunan Agricultural University.

## Author Contributions

XG and XM: conceptualization, methodology, and software. ZL, NL, XL, and FZ: literature collection. XG and JW: writing–original draft preparation. JC, QJ, and BT: writing–reviewing and editing. XM: funding acquisition. All authors contributed to the article and approved the submitted version.

## Conflict of Interest

The authors declare that the research was conducted in the absence of any commercial or financial relationships that could be construed as a potential conflict of interest.

## Publisher’s Note

All claims expressed in this article are solely those of the authors and do not necessarily represent those of their affiliated organizations, or those of the publisher, the editors and the reviewers. Any product that may be evaluated in this article, or claim that may be made by its manufacturer, is not guaranteed or endorsed by the publisher.

## Footnotes

^1^ http://rdp.cme.msu.edu/

## Abbreviations

BW: Body weight
ADG: Average daily gain
ADFI: Average daily feed intake
G:F: Gain to feed ratio
IgA: Immunoglobulin A
IgG: Immunoglobulin G
IgM: Immunoglobulin M
SOD: Superoxide dismutase
GSH-Px: Glutathione peroxidase
T-AOC: Total antioxidant capacity
MDA: Malondialdehyde
CAT: Catalase
AA: Amino acid
HPLC: High Performance Liquid Chromatography
AIA: Acid insoluble ash
AID: Apparent ileal digestibility
VFAs: Volatile fatty acids
SCFAs: Short chain fatty acids
BCFAs: Branched chain fatty acids
PCR: Polymerase chain reaction
OTUs: Operational taxonomic units
PCoA: Principal co-ordinates analysis
LDA: Linear discriminant analysis
LEfSe: Linear discriminant analysis effect size
PICRUSt: Phylogenetic investigation of communities by reconstruction of unobserved states
